# Cerebral Oxygen Saturation Responsiveness During Cardiopulmonary Resuscitation After Prolonged Ventricular Fibrillation: A Porcine Study

**DOI:** 10.3390/jcm15166289

**Published:** 2026-08-14

**Authors:** Yasuaki Koyama, Akira Ouchi, Nobutake Shimojo, Yoshiaki Inoue

**Affiliations:** 1 Department of Emergency and Critical Care Medicine, Hitachi General Hospital, 2-1-1, Jonan-cho, Hitachi 317-0077, Ibaraki, Japan; 2 Department of Adult Health Nursing, College of Nursing, Ibaraki Christian University, 6-11-1 Omika, Hitachi 319-1295, Ibaraki, Japan; 3 Department of Emergency and Critical Care Medicine, University of Tsukuba Hospital, 2-1-1 Amakubo, Tsukuba 305-8576, Ibaraki, Japan

**Keywords:** cardiogenic cardiac arrest, cerebral oxygenation, cerebral oxygen delivery, cerebral oxygen saturation, near-infrared spectroscopy, prolonged ventricular fibrillation, responsiveness

## Abstract

**Background/Objectives**: Near-infrared spectroscopy (NIRS) enables the monitoring of cerebral tissue oxygen saturation (ScO_2_) during cardiopulmonary resuscitation (CPR). In this study, we aimed to investigate ScO_2_ responsiveness during CPR initiated 14 min following the induction of ventricular fibrillation (VF) in a porcine model. **Methods**: Ten female pigs underwent electrically induced VF. CPR was initiated 14 min later using mechanical chest compressions. ScO_2_ was continuously monitored using NIRS. Return of spontaneous circulation (ROSC), movement recovery, and ScO_2_ dynamics were evaluated. **Results**: ScO_2_ reached its minimum value at 3.3 min (interquartile range [IQR], 2.8–4.3) following VF induction and remained suppressed for 10.8 min (IQR, 9.6–11.3) prior to CPR initiation. Although ScO_2_ reached its maximum value at 3.8 min (IQR, 2.8–4.3) following CPR initiation, the median ScO_2_ during CPR remained below 50% (49.0% [IQR, 48.3–50.5]). Initial mean arterial pressure during CPR was 35.5 mmHg (IQR, 31.5–40.3). Seven animals (70%) achieved ROSC, and three survived for 60 min post-ROSC; none of the animals recovered movement. **Conclusions**: Continuous assessment of ScO_2_ responsiveness using NIRS from the time of CPR initiation may provide useful physiological information regarding the restoration of cerebral oxygen delivery during resuscitation.

## 1. Introduction

In cardiogenic cardiac arrest (CA), approximately half of all initial lethal arrhythmias are ventricular fibrillation (VF) or ventricular tachycardia [1]. In Japan, among adult patients with out-of-hospital CA, only 6% exhibited VF upon emergency medical service arrival [2]. Furthermore, among adult (18–74 years) out-of-hospital patients with CA with an initial shockable rhythm upon emergency medical service arrival, only 34% remained shockable upon hospital arrival, with a 1-month survival rate of 36% and a favorable neurological outcome rate of 29% [3]. Thus, most patients with CA do not survive despite exhibiting a prolonged shockable rhythm upon hospital arrival. These findings suggest that physiological deterioration during prolonged VF influences resuscitation outcomes.

The management of VF is conceptualized in three time-dependent phases. During the electrical phase (from onset to approximately 4–7 min), immediate defibrillation is highly effective. During the circulatory phase (from approximately 4–7 to 10–17 min), cardiopulmonary resuscitation (CPR) is required to restore coronary and cerebral oxygen delivery before defibrillation. During the metabolic phase (beyond 10–17 min), targeted temperature management is necessary because of reperfusion injury [4,5,6]. Therefore, during the circulatory phase, physiological monitoring devices capable of estimating the elapsed time from CA onset and reflecting the adequacy of cerebral oxygenation and oxygen delivery remain necessary [4].

Near-infrared spectroscopy (NIRS) permits continuous, non-invasive monitoring of cerebral tissue oxygen saturation (ScO_2_) during CPR. Previous studies have reported relationships between cerebral oxygenation measured during resuscitation and ROSC or neurological outcomes [7,8,9,10]. In clinical practice, however, NIRS monitoring is often initiated after CPR initiation. Therefore, the interpretation of ScO_2_ responsiveness may be influenced by the duration for which CA remains untreated before monitoring initiation.

We previously compared changes in ScO_2_ between a VF-induced CA model (VF-CA group; cardiogenic), in which CPR was initiated 4 min after electrically induced VF, and an asphyxia CA model (A-CA group; respiratory), in which CA occurred after endotracheal tube clamping and CPR was initiated 4 min later [11]. ScO_2_ responsiveness during CPR was greater in the VF-CA group than in the A-CA group. However, because the two groups differed in both CA etiology and cerebral hypoxia duration before CPR, whether the impaired ScO_2_ responsiveness was primarily attributable to prolonged hypoxia or to the underlying mechanism of CA remains undetermined.

Therefore, we designed this study to isolate the physiological effect of prolonged cerebral hypoxia while maintaining the same arrest mechanism. We evaluated ScO_2_ dynamics in a porcine VF-induced CA model (VF-long-CA group) subjected to delayed CPR initiated 14 min after VF induction. We hypothesized that prolonged untreated VF would attenuate ScO_2_ responsiveness during CPR by impairing cerebral oxygen delivery despite VF being the initial rhythm.

## 2. Materials and Methods

The experimental protocol was reviewed and approved by the Institutional Animal Research Committee of our institution (protocol code 18-131). Animal care and experimental procedures complied with the National Institutes of Health Guidelines for the Care and Use of Laboratory Animals. The methods for drug administration, monitoring preparation, data collection, ventilator settings, and CPR procedures followed our previous protocol [11].

### 2.1. Animal Preparation

Ten 2-month-old female pigs were used as the VF-long-CA group. Sedation was induced with intramuscular ketamine (500 mg), followed by intravenous administration of propofol (loading, 1 mg/kg; maintenance, 4 mg/kg/h) and vecuronium (loading, 0.6 mg/kg; maintenance, 0.4–0.6 mg/kg/h). Following local anesthesia with 1% lidocaine, tracheostomy was performed and a 7 mm inner-diameter endotracheal tube was placed. Mechanical ventilation (MV) was provided with an LTV-1000 ventilator (CareFusion, San Diego, CA, USA) using an inspired oxygen fraction, 0.21; tidal volume, 10 mL/kg; positive end-expiratory pressure, 6 cmH_2_O; and a respiratory rate adjusted to maintain an end-tidal carbon dioxide (EtCO_2_) value of 40–45 mmHg. An arterial catheter was inserted into the femoral artery, and a 5-F pacing catheter was placed in the femoral vein. The positions of the endotracheal tube, pacing catheter, and heart were confirmed using a C-arm fluoroscopic X-ray system (DHF-105CX, Hitachi, Tokyo, Japan).

Cerebral oxygenation was assessed with a NIRO-200NX near-infrared spectroscopy monitor (Hamamatsu Photonics, Hamamatsu, Japan), with ScO_2_ expressed as the tissue oxygenation index (TOI). TOI is calculated as the percentage of oxygenated hemoglobin relative to total hemoglobin. A TOI value <50% is associated with cerebral ischemic intolerance [12], <55% with cerebral tissue damage [13], and ≥59% on hospital arrival after CA with survival to hospital discharge [14]. In clinical practice, however, NIRS sensors are generally placed bilaterally over the supraorbital regions to estimate cerebral oxygenation at a depth of approximately 2 cm [7]. In this animal study, probes were placed bilaterally over the temporal regions.

### 2.2. Study Design

Following calibration and synchronization of the monitoring systems, Ringer’s solution was administered rapidly. The animals were then observed for approximately 30 min to ensure stable baseline conditions without spontaneous movement or hemodynamic instability, after which baseline TOI, vital signs, and arterial blood gases were obtained.

**Figure 1:**
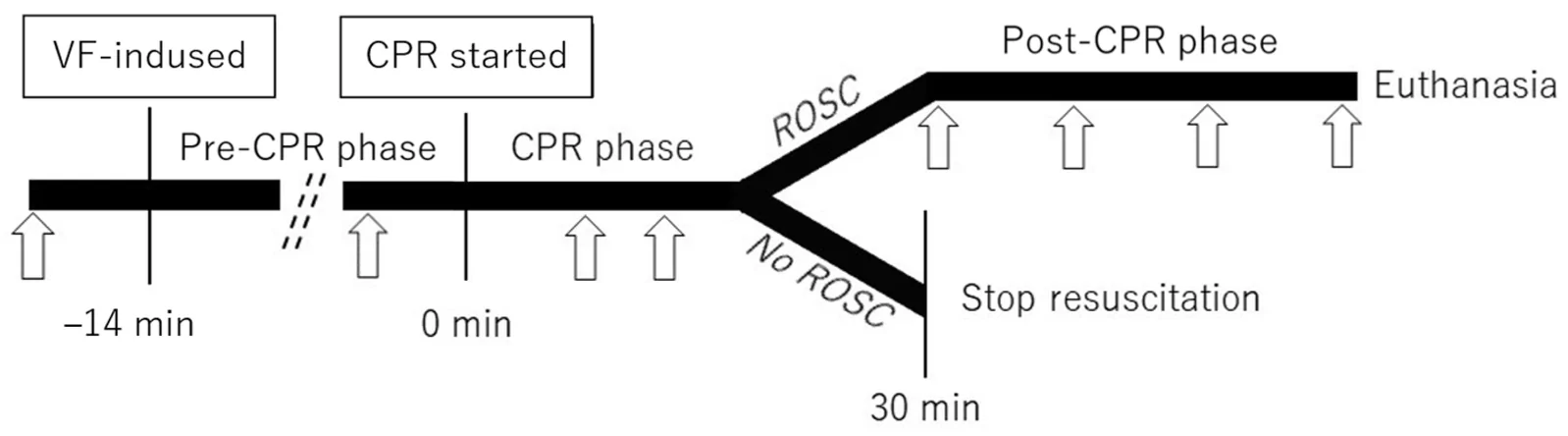
Timeline of the VF-long-CA experimental protocol. Arrows denote arterial blood gas sampling points: baseline, −1 min, and 3 min after CPR initiation; every 4 min thereafter; immediately after ROSC; and at 20 min intervals thereafter. VF, ventricular fibrillation; CPR, cardiopulmonary resuscitation; ROSC, return of spontaneous circulation.

VF was induced electrically through a pacing catheter connected to an electrical stimulator (GY600ATM; Kaifeng Huanan Equipment Co., Ltd., Kaifeng, China), after which MV was discontinued. CA duration was timed from induction, and CPR was started 14 min later (Pre-CPR phase; Figure 1). During the CA period, administration of propofol and vecuronium was discontinued, whereas Ringer’s solution infusion remained uninterrupted.

During the CPR phase, mechanical chest compression was performed with the LUCAS2™ system (Jolife, Lund, Sweden), with the compression point positioned over the center of the heart. MV was resumed with an inspired oxygen fraction, 1.0; tidal volume, 10 mL/kg; positive end-expiratory pressure, 6 cmH_2_O; and a respiratory rate, 10 breaths/min. Rhythm and pulse checks were performed at 2 min intervals. During the first 2 min, CPR consisted of asynchronous chest compressions and MV. Chest compressions were resumed within 10 s after each scheduled rhythm assessment and defibrillation in accordance with the ACLS (Advanced Cardiovascular Life Support) protocol. Thereafter, persistent VF was treated with 360-J monophasic defibrillation (Nihon Kohden, Tokyo, Japan) every 2 min and adrenaline (1 mg) every 4 min after the first defibrillation. If pulseless electrical activity (PEA) or asystole persisted, adrenaline was administered every 4 min after the initial 2 min of CPR. Infusion of Ringer’s solution was maintained, and antiarrhythmic agents were not administered, as previously described [15].

CPR was discontinued when ROSC was not achieved within 30 min of CPR initiation. After successful ROSC, rapid infusion of Ringer’s solution was continued during the post-CPR phase, and the inspired oxygen fraction was adjusted to maintain a peripheral oxygen saturation (SpO_2_) of 93–98%. Independent limb flexion and extension were regarded as signs of recovery of movement. Thereafter, propofol and vecuronium administration were resumed. Animals surviving for 60 min after ROSC were euthanized with potassium chloride.

### 2.3. Data Collection and Analysis

Physiological parameters, including heart rate, cardiac rhythm, arterial blood pressure (ABP), respiratory rate, SpO_2_, and EtCO_2_, were continuously recorded with a Philips IntelliVue MP50 monitor (Philips Medizin Systeme, Böblingen, Germany). ABG analyses were performed using either the EPOC (Alere, Waltham, MA, USA) or the ABL 735 (Radiometer Copenhagen, Copenhagen, Denmark). TOI measurements were recorded continuously at a sampling frequency of 1 Hz using bilateral cerebral probes. Hemodynamic measurements were available at 5 s intervals; therefore, TOI observations corresponding to these time points were selected for analysis. The selected TOI values were used without additional averaging.

All data are presented as medians (interquartile ranges [IQR]). Because of the limited sample size, subgroup analyses (ROSC vs. No-ROSC and VF vs. No-VF at CPR initiation) were considered exploratory and are presented descriptively without formal statistical testing. Statistical analyses were performed using EZR version 1.52 (Saitama Medical Center, Jichi Medical University, Saitama, Japan) [16], a graphical user interface designed for R software (The R Foundation for Statistical Computing, Vienna, Austria).

## 3. Results

### 3.1. Baseline

All animals remained free of CA during preparation. Median age was 68 days (67–72 days) and median body weight was 26.4 kg (25.8–27.0 kg). Throughout the experimental protocol, the total doses of propofol and vecuronium administered were 225.0 mg (185.0–247.5 mg) and 9.0 mg (8.3–10.8 mg), respectively, and the total volume of Ringer’s solution administered was 300.0 mL (250.0–380.0 mL).

### 3.2. Pre-CPR Phase

**Figure 2:**
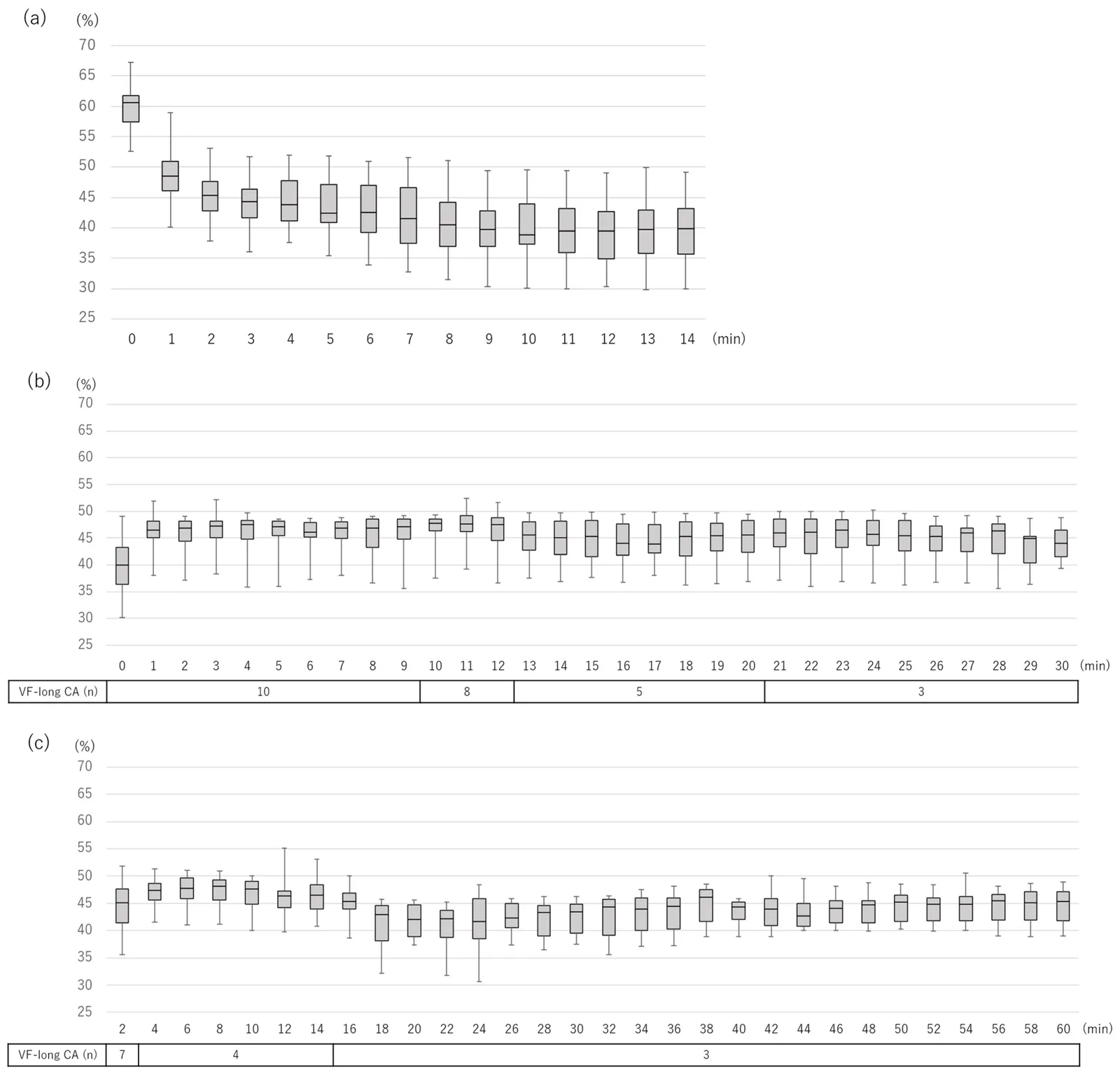
Changes in cerebral oxygen saturation (TOI) across the three experimental periods. (**a**) Pre-CPR phase, (**b**) CPR phase, and (**c**) Post-CPR phase. Data are shown as medians with interquartile ranges; CPR, cardiopulmonary resuscitation; VF-long-CA, ventricular fibrillation-long cardiac arrest.

TOI reached its minimum value at 3.3 min (2.8–4.3 min) following pre-CPR phase initiation, and the interval from the minimum TOI to CPR initiation was 10.8 min [9.6–11.3] (Figure 2a). Physiological parameters are presented in Table 1. At CPR initiation, the initial rhythms were VF in six animals, PEA in three, and asystole in one. 

### 3.3. CPR Phase

ROSC was achieved in seven of 10 animals (70%), including four with VF, two with PEA, and one with asystole. CPR duration was 16.0 min (12.0–27.5 min). CPR durations for the animals that achieved ROSC were 9, 12, 12, and 20 min for those with VF; 9 and 12 min for those with PEA; and 20 min for the one with asystole. The mean ABP (mABP) at CPR initiation was 35.5 mmHg (31.5–40.3 mmHg), and the mean value of mABP was 61.0 mmHg (53.5–77.8 mmHg) (Table 1).

Figure 2b depicts the TOI changes (maximum TOI, 49.0% [48.3–50.5%]; median TOI, 46.6% [43.2–48.1%]). The time from CPR initiation to the maximum TOI achievement was 3.8 min (2.8–4.3 min) (Table 1). Figure S1 depicts the TOI changes in the ROSC and No-ROSC groups in the CPR phase.

As in our previous study [11], the singular point was defined as the time point showing the maximum positive change in TOI between consecutive 5 s measurements before the maximum TOI value was reached. The time from CPR initiation to the singular point was 0.6 min (0.3–2.0 min), and the TOI velocity from CPR initiation to the singular point was 8.4%/min (1.9–18.0%/min) (Table 1).

In the subgroups, the time from CPR initiation to the maximum TOI attainment and to the singular point appeared to be shorter in the ROSC group (Table S1). The TOI velocity from CPR initiation to the singular point was 15.7%/min (6.9–20.4%/min) in the ROSC group, 1.8%/min (0.5–2.3%/min) in the No-ROSC group, 13.1%/min (1.6–18.0%/min) in the VF group, and 5.3%/min (2.5–24.6%/min) in the No-VF group.

### 3.4. Post-CPR Phase

Post-ROSC survival time was 16 min (3.5–60 min). Three animals (one with VF and two with PEA) survived for 60 min post-ROSC. None of the animals recovered their movement. Figure 2c exhibits the post-ROSC TOI values. The interval from ROSC to the maximum TOI attainment was 1.2 min (0.9–7.6 min).

## 4. Discussion

When delayed CPR was initiated 14 min following VF induction (VF-long-CA group), ScO_2_ reached its minimum value at approximately 3.3 min after VF induction, remained suppressed throughout the Pre-CPR phase, and increased after CPR initiation, reaching its maximum value at approximately 4 min later. However, the median ScO_2_ remained below 50% throughout CPR despite an ROSC rate of 70%. None of the animals recovered movement. The principal finding of the present study is that even when the initial rhythm was VF, prolongation of untreated VF attenuated ScO_2_ responsiveness during CPR. This finding extends our previous observations and supports the hypothesis that prolonged untreated VF impairs restoration of cerebral oxygen delivery during resuscitation.

None of the pigs in the VF-long-CA group recovered movement despite a 70% ROSC rate. This clinical course was similar to that observed in the A-CA group in our previous study [11], in which CA occurred approximately 10 min after endotracheal tube clamping. CPR was initiated after 4 min, yielding a total cerebral hypoxia duration of approximately 14 min. Furthermore, previous animal studies comparing VF and asphyxial CA have suggested that neurological injury may be influenced by the duration of cerebral hypoxia. In a canine study [17], CPR was initiated 10 min after electrically induced VF and 7 min after CA onset in the asphyxial group. Although the ROSC rate was 84% in the VF group and 100% in the asphyxial group, neurological impairment was similar between groups. However, since CA occurred approximately 8 min after tube clamping, the comparison might have been between a VF group with 10 min of cerebral hypoxia and an asphyxial group with approximately 15 min of cerebral hypoxia. In a porcine study [15], a VF group underwent CPR 8 min after VF induction and an asphyxial group underwent CPR 8 min after CA onset. The ROSC rate was 94% in the VF group and 61% in the asphyxial group, whereas neurological outcome and cerebral metabolic injury were worse in the asphyxial group. Because the interval from tube clamping to CA onset was not reported, the actual cerebral hypoxia duration in the asphyxial group may have been considerably longer. However, these comparisons should be interpreted cautiously because they involved different animal species and experimental protocols. Nevertheless, together with our findings, they support the concept that prolonged cerebral hypoxia, irrespective of the initial arrest rhythm, is associated with impaired cerebral physiological recovery during resuscitation.

In the VF-long-CA group, ScO_2_ reached the minimum value at 3.3 min following VF induction, remained suppressed for approximately 10 min, and increased after CPR initiation. Although ScO_2_ reached its maximum value at approximately 4 min following CPR initiation, the median ScO_2_ remained below 50%, similar to the previous findings in the A-CA group [11]. Before CPR, arterial oxygenation remained relatively preserved in the VF-long-CA group because MV had been discontinued after VF induction, whereas severe hypoxemia developed in the A-CA model owing to airway obstruction. However, despite similar pre-CPR PaO_2_ values between the present VF-long-CA group and the previously reported VF-CA group, the initial mABP immediately following CPR initiation was 35.5 mmHg in the VF-long-CA group and 69.5 mmHg in the previously reported VF-CA group. Prolonged untreated VF may have resulted in severe myocardial dysfunction, thereby limiting restoration of systemic perfusion at CPR onset [18]. Consequently, cerebral oxygen delivery may have been impaired during CPR despite preserved arterial oxygenation before CPR, which may explain the attenuated ScO_2_ responsiveness observed in the present study. This interpretation is consistent with the physiological determinants of ScO_2_, which depend not only on arterial oxygen content but also on cerebral perfusion and oxygen extraction [19]. Thus, persistent impairment of systemic and cerebral perfusion after prolonged untreated VF may have limited the recovery of cerebral oxygenation throughout the CPR period.

The ROSC group tended to show a more rapid increase in ScO_2_ than the No-ROSC group. In our previous study [11], the TOI velocity in the VF-CA group, in which 90% of the group achieved ROSC, was 16.6 (5.5–32.6) %/min, comparable to that observed in the ROSC group in the present study (15.7 [6.9–20.4] %/min). Because all animals underwent standardized mechanical CPR using the same protocol, with chest compressions delivered by the LUCAS2 device and interruptions minimized according to ACLS recommendations, differences in ScO_2_ responsiveness are unlikely to be attributable to major differences in CPR delivery itself. Both ROSC and No-ROSC groups were exposed to similar durations of cerebral hypoxia before CPR. In contrast, mABP, EtCO_2_, and PaO_2_ tended to be higher in the ROSC group, suggesting that more effective restoration of cerebral oxygen delivery contributed to a more rapid increase in ScO_2_. Similarly, a porcine resuscitation study using INVOS after 10 min of untreated VF demonstrated rapid increases in cerebral oxygen saturation among animals achieving ROSC [20]. These observations are consistent with the physiological requirement for adequate coronary and systemic perfusion during successful resuscitation [21]. Therefore, the observed ScO_2_ responsiveness may reflect physiological recovery during CPR; however, the present study was not designed to assess its association with subsequent ROSC or neurological outcomes.

Although the ROSC group tended to exhibit a rapid increase in ScO_2_, ScO_2_ remained below 50% throughout CPR, and no animals recovered movement. In contrast, in our previous study [11], the median ScO_2_ during CPR in the VF-CA group remained above 50%, and 70% of animals recovered movement. Studies have demonstrated that maintenance of cerebral perfusion is essential for neurological recovery following CA [22,23,24]. However, because neurological assessment in the present study was limited to movement recovery during the first 60 min after ROSC, these findings do not establish ScO_2_ responsiveness as a predictor for neurological outcomes. Rather, they suggest that sustained impairment of cerebral oxygenation during CPR accompanies severe cerebral injury after prolonged untreated VF.

NIRS monitoring facilitates the assessment of cerebral hypoxia, and in many clinical settings, NIRS monitors are applied during CPR. However, the clinical significance of isolated initial (at the time of device placement), maximum, minimum, or mean ScO_2_ values remains uncertain [25]. The present findings emphasize the value of continuous monitoring of ScO_2_ responsiveness starting at CPR initiation to characterize physiological changes in cerebral oxygenation during CPR. Furthermore, they demonstrate that prolonged untreated VF can attenuate ScO_2_ responsiveness despite VF being the initial rhythm, suggesting that ScO_2_ responsiveness reflects the physiological consequences of prolonged ischemia.

This study had some limitations. First, this study was conducted as an additional exploratory experiment and was not designed for direct statistical comparison with the VF-CA and A-CA groups from our previous study [11]. In addition, due to the limited sample size, subgroup analyses were descriptive, and formal statistical comparisons could not be performed. Second, neurological evaluation was limited to recovery of spontaneous movement during the first 60 min after ROSC. Standardized neurological deficit scoring, histopathological evaluation, and long-term neurological assessment were not performed. In addition, residual effects of vecuronium after prolonged CA may have influenced movement recovery. Therefore, the present findings do not validate ScO_2_ responsiveness as a neurological prognostic marker. Third, the 14 min untreated VF interval was selected to approximate the cerebral hypoxia duration previously observed in our A-CA model [11]. However, the present experiment was not designed to determine the longest duration of untreated VF compatible with neurological recovery. Previous porcine studies have reported favorable neurological recovery with CPR initiation after approximately 13 min of untreated VF but poor neurological outcomes with delayed CPR to 15 min [26,27]. Another porcine study demonstrated a very low likelihood of ROSC after untreated VF exceeded 20 min despite extracorporeal support [28]. Finally, because this was an animal study, further clinical investigations are required to determine whether similar physiological responses are observed in humans and whether interventions that improve ScO_2_ responsiveness also improve clinical outcomes.

## 5. Conclusions

When CPR was initiated 14 min after VF induction, ScO_2_ reached its minimum value at 3.3 min following VF induction and remained suppressed for 10.8 min prior to CPR initiation. Although ScO2 reached its maximum value at 3.8 min following CPR initiation, the median ScO2 during CPR remained below 50%. Although 70% of animals achieved ROSC, none of them recovered movement during the observation period. These findings suggest that prolonged untreated VF attenuates ScO_2_ responsiveness during CPR. Continuous assessment of ScO_2_ responsiveness using NIRS from CPR initiation may provide useful physiological information regarding the restoration of cerebral oxygen delivery during resuscitation. However, these findings are limited to this experimental porcine model and should be validated in future clinical studies.

## Figures and Tables

**Table 1:** Comparison of parameters for each phase.

	Baseline (*n* = 10)	Pre-CPR Phase (*n* = 10)	CPR Phase (*n* = 10)	Post-CPR Phase (*n* = 7)
mABP initial (mmHg)	-	-	35.5 [31.5–40.3]	81.0 [51.0–152.5]
mABP median (mmHg)	91.1 [84.2–99.8]	32.0 [29.5–35.5]	61.0 [53.5–77.8]	39.0 [36.5–56.5]
EtCO_2_ median (mmHg)	45.1 [42.3–47.8]	–	31.0 [20.5–34.0]	13.0 [9.5–35.5]
pH	7.44 [7.42–7.47]	7.55 [7.50–7.57]	7.14 [7.10–7.19]	7.06 [7.06–7.10]
PaO_2_ (mmHg)	194.6 [157.5–217.3]	78.1 [74.8–83.4]	88.9 [70.8–269.5]	153.5 [123.2–249.0]
PaCO_2_ (mmHg)	42.6 [40.1–46.4]	26.4 [22.5–28.5]	58.0 [46.5–67.3]	40.1 [33.7–51.9]
Lactate (mmol/L)	2.85 [2.30–3.63]	4.3 [3.3–5.8]	11.2 [10.9–12.9]	13.8 [13.2–17.5]
TOI (%) Initial	–	60.6 [57.4–61.8]	40.0 [36.4–43.2]	46.4 [42.2–47.6]
Maximum	–	60.6 [57.6–61.8]	49.0 [48.3–50.5]	47.3 [43.7–52.5]
Minimum	–	36.3 [32.3–39.5]	39.1 [35.1–42.2]	43.1 [38.7–46.7]
Median	–	41.3 [39.4–45.3]	46.6 [43.2–48.1]	42.3 [40.1–46.2]
Value at the singular point	–	–	44.0 [41.5–48.0]	–
Time to the singular point (min)	–	–	0.6 [0.3–2.0]	–
Time to Maximum TOI (min)	–	–	3.8 [2.8–4.3]	–
TOI velocity to the singular point (%/min)	–	–	8.4 [1.9–18.0]	–

Data are presented as medians [interquartile ranges]. CPR, cardiopulmonary resuscitation; mABP, mean arterial blood pressure; EtCO_2_, end-tidal CO_2_; PaO_2_, partial pressure of arterial oxygen; PaCO_2_, partial pressure of arterial carbon dioxide; TOI, tissue oxygenation index.

## Data Availability

The data that support the findings of this study are available from the corresponding author, Y.K., upon reasonable request.

## Supplementary Materials

The following supporting information can be downloaded at https://www.mdpi.com/article/10.3390/jcm15166289/s1. Table S1: Parameters according to ROSC outcome and rhythm status; Figure S1: Changes in cerebral oxygen saturation (tissue oxygenation index [TOI]) in ROSC and No-ROSC group in the CPR phase.
