# Supplementary material for: Cerebral Oxygen Saturation Responsiveness During Cardiopulmonary Resuscitation After Prolonged Ventricular Fibrillation: A Porcine Study
Source: J Clin Med. 2026 Aug 14;15(16):6289. doi: 10.3390/jcm15166289 (PMC13513912; doi:10.3390/jcm15166289)
Supplement: Supplementary file 1 [file jcm-15-06289-s001.zip › jcm-4407140-supplementary.pdf]

Supplementary Table S1. Parameters according to ROSC outcome and rhythm status.

CPR Phase	ROSC (n = 7)	No-ROSC (n = 3)	VF (n = 6)	No-VF (n = 4)
TOI (%) Initial	38.4 [35.3–41.0]	43.6 [40.9–46.3]	40.0 [36.5–43.2]	39.4 [36.1–42.3]
Maximum	48.8 [48.2–49.6]	50.0 [48.7–50.7]	49.1 [48.5–50.5]	48.7 [47.9–50.0]
Minimum	38.0 [34.8–40.2]	41.5 [37.0–42.8]	39.1 [35.5–42.2]	38.5 [34.8–41.0]
Median	46.1 [43.0–47.9]	46.8 [44.2–48.3]	46.6 [44.2–47.9]	44.9 [42.5–48.1]
Value at the singular point	43.4 [40.6–46.9]	48.2 [47.1–50.0]	45.6 [43.0–48.0]	42.9 [39.6–48.2]
Time to the singular point (min)	0.3 [0.1–0.6]	3.8 [2.7–4.1]	0.7 [0.2–3.7]	0.5 [0.3–1.8]
Time to Maximum TOI (min)	3.4 [2.5–3.8]	6.5 [4.6–7.6]	4.0 [3.6–4.6]	2.8 [2.7–3.7]
TOI velocity to the singular point (%/min)	15.7 [6.9–20.4]	1.8 [0.5–2.3]	13.1 [1.6–18.0]	5.3 [2.5–24.6]
mABP initial (mmHg)	38.0 [32.0–40.5]	33.0 [30.0–37.0]	39.5 [32.8–42.5]	33.0 [32.5–34.3]
mABP median (mmHg)	74.0 [56.5–81.5]	51.0 [47.5–57.5]	59.5 [53.5–79.0]	66.0 [54.5–75.3]
$EtCO_2$ median (mmHg)	32.0 [26.0–34.0]	11.0 [11.0–24.0]	34.0 [23.5–34.0]	26.0 [19.3–30.5]
pH	7.15 [7.11–7.19]	7.14 [7.11–7.24]	7.14 [7.09–7.15]	7.22 [7.16–7.27]
PaO_2 (mmHg)	95.9 [80.0–269.0]	68.4 [41.7–174.0]	80.0 [70.8–92.4]	274.9 [219.3–293.1]
$PaCO_2$ (mmHg)	60.2 [47.6–66.3]	55.8 [43.2–72.8]	62.2 [56.9–67.3]	41.8 [36.2–53.2]

Lactate (mmol/L)	12.9 [11.2–13.2]	10.9 [9.9–11.0]	11.2 [11.0–12.5]	11.9 [10.9–13.6]

Data are presented as medians [interquartile ranges]. Subgroup data are presented descriptively because of the limited sample size. CPR, cardiopulmonary resuscitation; mABP, mean arterial blood pressure; $EtCO_2$, end-tidal CO_2; PaO_2, partial pressure of arterial oxygen; $PaCO_2$, partial pressure of arterial carbon dioxide; TOI, tissue oxygenation index; ROSC, return of spontaneous circulation; VF, ventricular fibrillation.

Supplementary Figure S1. Changes in cerebral oxygen saturation (tissue oxygenation index [TOI]) in ROSC and No-ROSC group in the CPR phase.

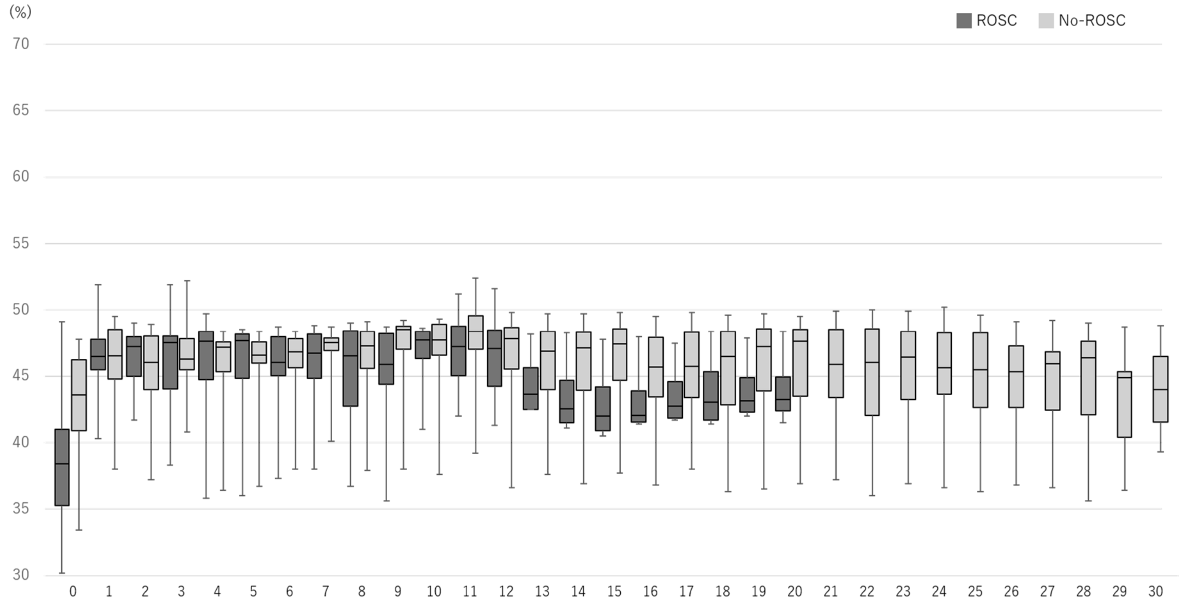

ROSC (n)	7	5	2	0
No-ROSC (n)	3			

TOI values are presented as medians (interquartile ranges); CPR, cardiopulmonary resuscitation; ROSC, return of spontaneous circulation; VF, ventricular fibrillation.
